# The role of ethylene and ROS in salinity, heavy metal, and flooding responses in rice

**DOI:** 10.3389/fpls.2014.00685

**Published:** 2014-12-04

**Authors:** Bianka Steffens

**Affiliations:** Department of Plant Physiology, Faculty of Biology, Philipps University, Marburg, Germany

**Keywords:** abiotic stress response, chromium, ethylene, flooding, reactive oxygen species, rice, salt

## Abstract

Plant growth and developmental processes as well as abiotic and biotic stress adaptations are regulated by small endogenous signaling molecules. Among these, phytohormones such as the gaseous alkene ethylene and reactive oxygen species (ROS) play an important role in mediating numerous specific growth or cell death responses. While apoplastic ROS are generated by plasma membrane-located respiratory burst oxidase homolog proteins, intracellular ROS are produced mainly in electron transfer chains of mitochondria and chloroplasts. Ethylene accumulates in plants due to physical entrapment or by enhanced ethylene biosynthesis. A major crop that must endure high salt and heavy metal concentrations upon flooding in regions of Asia is rice. Ethylene and ROS have been identified as the major signals that mediate salinity, chromium, and flooding stress in rice. This mini review focuses on (i) what is known about ethylene and ROS level control during these abiotic stresses in rice, (ii) how the two signals mediate growth or death processes, and (iii) feedback mechanisms that in turn regulate ethylene and ROS signaling.

## INTRODUCTION

Flooding is a major abiotic stress which results in crop yield losses in a wide range of different landscapes. Some crop species can endure soil waterlogging for some hours while other flood tolerant crops can cope with partial or complete flooding for some days or months (Bailey-Serres and Voesenek, 2008). In addition to flooding, salt concentrations may rise when salts delivered by water from the ocean or from flushing out salts from wet soil. In addition, heavy metal availability may change due to changing pH values upon flooding. High salinity and heavy metal concentrations can similarly reduce crop growth and may even cause plant death (e.g., Li et al., 2014; Trinh et al., 2014).

Flood tolerant crops possess anatomical, metabolic, or morphological adaptations. One metabolic response to flooding, heavy metals and high salinity, is the induction of ethylene production (Kende, 1993; Li et al., 2014; Trinh et al., 2014). Ethylene production is enhanced in crops such as barley and rice (Kende, 1993; Vassilev et al., 2004; Li et al., 2014). Only tolerant rice plants, however, endure longer phases of soil waterlogging or submergence (Bailey-Serres and Voesenek, 2008). Ethylene is produced in a two-step reaction. The first specific step is the formation of 1-aminocyclopropane-1-carboxylate (ACC) by ACC synthase (ACS), the second step implies ethylene formation from ACC by ACC oxidase (ACO; Kende, 1993). Ethylene is not only produced during these abiotic stresses, but has also been identified as regulator of stress-related morphological responses such as primary and secondary root growth or aerenchyma formation in internodes, roots, and leaves (e.g., Parlanti et al., 2011; Steffens et al., 2011). In *Arabidopsis*, ethylene is perceived by five ER-localized two-component receptors such as ETR1 (ETHYLENE RESISTANT1; Chen et al., 2002). Downstream of ethylene receptors the signaling cascade consists, of a member of the Nramp family of ion transporters, EIN2 (ETHYLENE INSENSITIVE2), of transcription factors such as EIN3 and of members of the APETALA2/ETHYLENE RESPONSE FACTOR (AP2/ERF) multi gene family.

Reactive oxygen species (ROS) have been identified as a second class of small molecules that mediate responses to flooding, heavy metals, and high salinity. ROS are generated from molecular oxygen and their origin is diverse. Apoplastic ROS are generated through plasma membrane-located Rboh (respiratory burst oxidase homolog) proteins. Rboh proteins in plants are homologs of mammalian NADPH oxidase subunit gp91^phox^ (Torres et al., 1998) that produce superoxide anions (${\text{O}}_{2}^{•-}$). Short-lived ${\text{O}}_{2}^{•-}$ dismutate to the non-radical hydrogen peroxide (H_2_O_2_) either spontaneously, or catalyzed by superoxide dismutase (SOD) or ascorbate peroxidases (POD). In addition, ${\text{O}}_{2}^{•-}$, hydroxyl radicals, hydroperoxyl radicals, ozone, and singlet oxygen are produced through the reductive power provided by electron transport chains of mitochondria and chloroplasts and through peroxisomal activity (Blokhina and Fagerstedt, 2010; Chang et al., 2012). Non-enzymatic ROS scavenging proteins such as cysteine-rich metallothioneins (MTs) as well as antioxidant enzymes, e.g., SOD, catalase, and glutathione reductase (GR) are essential for ROS homeostasis. During biotic and abiotic stress, the cellular ROS balance is disturbed by either enhancing ROS generation or reducing ROS scavenging abilities (Steffens et al., 2013).

Plants react to different incoming signals when flooding, high salinity, and heavy metal stress occur in combination. The plants’ ability to coordinate these signals and start the adaptive survival responses requires mainly two internal signals: ethylene and different ROS. This review focuses on ethylene and ROS as signaling intermediates in salinity, chromium, and flooding stress responses in the crop species rice.

## MAPK-MEDIATED PHOSPHORYLATION AFFECTS ETHYLENE AND ROS HOMEOSTASIS DURING SALT STRESS SIGNALING

In all other plant species than halophytes, high sodium chloride concentrations cause growth retardation and may result in plant death because of drastic changes in ion and ROS homeostasis and in altered gene expression (Li et al., 2014). In addition, salt stress induces ethylene generation, ethylene can function as a downstream signal and alter gene expression as well (Wang et al., 2002). Interestingly, enhanced synthesis of ACC, the natural precursor of ethylene, seems to reduce salt tolerance (Dong et al., 2011). Ethylene signaling in turn is required for salinity tolerance of plants (Dong et al., 2011), showing the importance of ethylene homeostasis during salt stress.

Lectin receptor-like kinases (RLKs) are a family with 173 members in rice. The lectin RLK protein consist of an N-terminal lectin, a transmembrane domain, and a C-terminal kinase domain (Vaid et al., 2012). They are involved in developmental processes, in biotic stress signaling or in self-incompatibility (Vaid et al., 2012). Like other RLKs, lectin RLKs mediate the incoming signals through phosphorylation of mitogen-activated protein kinases (MAPKs). Activation of MAPK cascade signaling and enhanced ROS generation are further salt stress responses (Kiegerl et al., 2000; Teige et al., 2004).

**FIGURE 1:**
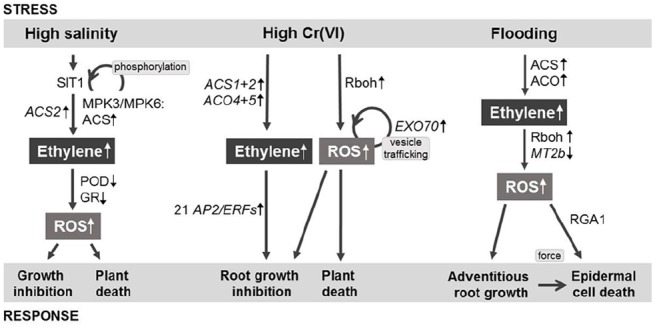
**Simplified model of salinity-, chromium-, and flooding-induced signaling pathways in rice.** Ethylene and ROS are the major internal signals. Plant responses comprise growth regulation and death. Other hormones and signals are not shown. Abbreviations and details are described in the text (based on Steffens et al., 2012; Li et al., 2014; Trinh et al., 2014).

The plasma membrane-located lectin RLK SIT1 (SALT INTOLERANCE1) is mainly located at the surface of rice root cells (Li et al., 2014). SIT1 is activated under high salinity conditions and mediates salt stress signaling through direct phosphorylation of OsMPK3 and OsMPK6 (Figure 1). Activated OsMPK3 and OsMPK6 in turn phosphorylate ACS proteins. Phosphorylation of ACS proteins results in increased protein stability and activity, hence in enhanced ethylene production. SIT1 upregulates *ACS2* during salt stress, pointing to an additional transcriptional regulation of genes relevant for ethylene biosynthesis. Ethylene signaling through ETR1, EIN2, and EIN3 is also part of the SIT1 signaling pathway during salt stress. During salt stress, OsMPK3 and OsMPK6 activities act upstream of ethylene signaling (Li et al., 2014). Ethylene was shown to be required for ROS accumulation upon salt stress (Li et al., 2014). ROS accumulation is dependent on reduced POD and GR activities (Li et al., 2014).

Ethylene responsive transcription factors of other species have been described to be involved in the control of ROS generation and signaling. Transcription factors of the AP2/ERF multi gene family link the signaling pathways of ROS and ethylene during abiotic stress. In *Tamarix hispida*, ERF1 inhibits the expression of SOD and POD genes during drought or high salinity, thus leading to enhanced ROS levels due to reduced scavenging ability (Wang et al., 2014). In rice, this link has not yet been analyzed.

## ETHYLENE, ROS, AND VESICLE TRAFFICKING—TEAM PLAYERS DURING CHROMIUM STRESS IN RICE

A side effect of waterlogging or flooding may be the pH-dependent increased availability of the heavy metal chromium from soils. Principal component analysis of different data sets from soil studies in Asia suggested that the concentration of chromium correlated with the soil pH values (Zarcinas et al., 2004). Toxicity of heavy metals is based on (i) exchange of required ions from protein binding sites, (ii) cellular redox perturbation through altering ROS production and scavenging, and (iii) membrane damage; toxicity often depends (iv) on the valence state of the heavy metal ion (Sharma and Dietz, 2009). Copper and chromium are merely two redox-active heavy metals that at high concentrations in soil induce oxidative stress and hence growth retardation in plants such as pea (Palma et al., 1987) or red cabbage (Posmyk et al., 2008). Upon heavy metal stress ROS are produced mainly through electron transport chains of mitochondria and chloroplasts and through peroxisomal activity (Dixit et al., 2002; Pandey et al., 2009). In addition, Rboh contribute to apoplastic ROS generation induced by heavy metals (Trinh et al., 2014).

Induction of oxidative stress by the most toxic form of chromium Cr(VI) was identified as the major problem for seedling growth in rice (Panda, 2007; Zeng et al., 2012). Cr stress resulted in ROS generation through plasma membrane-located Rboh (Trinh et al., 2014) and elevated lipid peroxidation (Panda, 2007). Based on transcriptome profiles of rice seedling roots that were obtained after 1 and 3 h of application of Cr(VI), ethylene biosynthesis and signaling, vesicle trafficking and ROS level modulation were identified as being part of the Cr signaling pathway (Huang et al., 2014; Trinh et al., 2014; Figure 1).

Various plant hormone increase upon different heavy metal stresses. During high cadmium concentrations in pea and barley ethylene levels are elevated (Vassilev et al., 2004; Rodríguez-Serrano et al., 2006). Upregulation of two of the six ACS gene family members, *ACS1* and *ACS2*, and of two of the seven ACO gene family members in rice, *ACO4* and *ACO5* (Rzewuski and Sauter, 2008), indicates that ethylene synthesis is part of Cr signaling (Trinh et al., 2014). In rice, *ACS1* is also induced by hypoxia, anoxia, ethylene, and H_2_O_2_ (Zarembinski and Theologis, 1993; Steffens and Sauter, 2009a), and *ACS2* and *ACO5* are both induced by infection with the fungus *Magnaporthe grisea* (Iwai et al., 2006), linking these genes to ethylene biosynthesis upon abiotic and biotic stress. Ethylene, together with other plant hormones may contribute to growth inhibition during Cr stress.

Upregulation of more than twofold of AP2/ERF genes by Cr treatment was observed in 21 members of different gene family subgroups and downregulation of a member of subgroup Ib, *ERF120* (Nakano et al., 2006; Trinh et al., 2014). Three genes, namely *ERF67* (subgroup VIIa), *ERF68* (subgroup VIIa), and *ERF77* (subgroup VIIIa), were upregulated by Cr and drought in rice (Nakano et al., 2006; Wang et al., 2011; Trinh et al., 2014). In addition, subgroup VII AP2/ERFs such as *SNORKEL1* (*SK1*), *SNORKEL2* (*SK2*), and *SUBMERGENCE1A-1* (*SUB1A-1*) were previously identified as being crucial for ethylene-mediated growth regulation of rice upon submergence (Fukao et al., 2006; Hattori et al., 2009). Hence, *ERF67*, *ERF68*, and *ERF77* are subject to regulation by different abiotic stresses, indicating that they may be general abiotic stress response genes.

Vesicle trafficking contributes to exocytosis, hence cell growth and other adaptive reactions of plants to stresses are affected. Exocyst subunit Exo70 is part of a protein complex with eight subunits that mediates vesicle trafficking from the post-Golgi to the plasma membrane. Upregulation upon Cr treatment of five Exo70 genes of different groups of the 41 Exo70 gene family members in rice indicates that exocytosis contributes to early Cr signaling (Trinh et al., 2014). Vesicle trafficking-associated gene expression was also found in roots of *Salix* during Cr(VI) stress (Quaggiotti et al., 2007). Inhibition of vesicle trafficking by pharmacological approaches using brefeldin A resulted in reduced ROS generation during Cr(VI) and Cu stress, indicating that vesicle trafficking mediated by the exocyst complex increases oxidative stress during heavy metal stress (Lin et al., 2013; Trinh et al., 2014). The involvement of vesicle trafficking itself or vesicle trafficking-associated gene expression have been, to my knowledge, not analyzed yet for the signaling pathway or biosynthesis.

## ETHYLENE, ROS, AND MECHANICAL SIGNALING MEDIATE EPIDERMAL CELL DEATH UPON FLOODING IN RICE

Morphological adaptive responses of submerged rice plants, such as internodal growth or spatially controlled death of parenchymal cells during aerenchyma formation in various organs, are regulated through ethylene, abscisic acid, and/or gibberellin signaling (e.g., Raskin and Kende, 1984a,b; Justin and Armstrong, 1991; Hoffmann-Benning and Kende, 1992; Steffens et al., 2011). The gaseous hormone ethylene accumulates upon submergence due to physical entrapment and enhanced biosynthesis. Ethylene is accepted to be the major regulator. In deepwater rice, ethylene helps the foliage to escape submergence by fast stem growth mediated by *SK1* and *SK2* (Hattori et al., 2009). In flooding-resistant cultivars ethylene inhibits stem growth during the quiescence response regulated by another subgroup VII AP2/ERF transcription factor, *SUB1A-1* (Xu et al., 2006). The involvement of subgroup VII AP2/ERFs in aerenchyma formation in internodes, primary and secondary roots is largely unknown. Interestingly, analysis of aerenchyma formation in leaf sheaths of a variety where *Sub1A* is absent and a variety containing the *Sub1A* gene revealed that either ethylene accumulation and signaling or ROS signaling are important (Parlanti et al., 2011).

Furthermore, ethylene mediates submergence-induced growth of adventitious roots (Lorbiecke and Sauter, 1999; Steffens et al., 2012) and death of the epidermal cells overlying adventitious root primordia (Mergemann and Sauter, 2000; Steffens and Sauter, 2009a; Steffens et al., 2012). These ethylene-regulated responses are mediated by both ROS accumulation and/or ROS signaling. Transcriptome analysis revealed that *ACO1* is upregulated in epidermal cells overlying adventitious root primordia, hence locally increased ACO1 activity could result in spatial control of ethylene biosynthesis (Mekhedov and Kende, 1996; Steffens and Sauter, 2009a). Death of epidermal cells overlying adventitious root primordia depends on Rboh activity, ethylene and in addition on a mechanical signal. Force generated by the growing adventitious root primordium combined with chemical signaling results in locally induced epidermal cell death (Steffens et al., 2012; Figure 1). Epidermal cell death is furthermore mediated by RGA1 (rice heterotrimeric G protein alpha subunit; D1) that encodes the single Gα subunit of heterotrimeric G protein in rice (Steffens and Sauter, 2009b). G protein signaling is an essential part of epidermal cell death. *d*1 mutant plants that have a repressed expression of RGA1 showed a reduction of epidermal cell death in response to ethylene and H_2_O_2_. The role of heterotrimeric G protein in salinity and heavy metal stress has not been analyzed yet in rice.

## ETHYLENE AND ROS—SMALL MOLECULES AND THEIR COMPLEXITY

Various abiotic stresses occur during a plants’ life due to its sessile way of life. Crops like rice have to cope with high levels of salt and soil contaminations such as chromium that occur with rising water levels. Responses to these tremendous stresses are mediated by ethylene and ROS which act as internal signals. Ethylene and ROS are intermediators of gene expression as far as growth regulation or specific cell death are affected. Sometimes the plant dies due to these stresses, but processes triggered by ethylene and ROS often prevent plants’ death. Besides the chemical signals, phosphorylation cascades, vesicle trafficking, or mechanosignaling were identified as being part of salinity, chromium, or submergence signaling, respectively. These and other results not mentioned here pinpoint to the complexity of ethylene and ROS signaling upon abiotic stress responses. A closer look into ethylene signaling and ROS homeostasis in the future will help to itemize the regulatory network that leads to plant survival responses upon abiotic stresses.

### Conflict of Interest Statement

The author declares that the research was conducted in the absence of any commercial or financial relationships that could be construed as a potential conflict of interest.
